# “Bariatric Beriberi”: A Rare Case of Wernicke Encephalopathy Two Weeks After Laparoscopic Sleeve Gastrectomy

**DOI:** 10.7759/cureus.37056

**Published:** 2023-04-03

**Authors:** Lefika Bathobakae, Sacide S Ozgur, Danielle Lombardo, Nader Mekheal, Patrick Michael

**Affiliations:** 1 Internal Medicine, St. Joseph's Regional Medical Center, Paterson, USA; 2 Medical Education, St. George's University School of Medicine, St. Georgestown, GRD; 3 Internal Medicine, St. Joseph Hospital and Medical Center, Paterson, USA; 4 Internal Medicine, St. Joseph's Univeristy Medical Center, Paterson, USA

**Keywords:** morbid obesity, previous sleeve gastrectomy, thiamine, wernicke encephalopathy, beriberi

## Abstract

Wernicke encephalopathy (WE) is an acute neurological syndrome caused by thiamine (vitamin B1) deficiency. This disorder manifests as a triad of gait ataxia, confusion, and vision abnormalities. The absence of a full triad does not rule out WE. Because of its vague presentation, WE is commonly missed in patients with no history of alcohol abuse. Other risk factors for WE include bariatric surgery, hemodialysis, hyperemesis gravidarum, and malabsorption syndromes. WE is a clinical diagnosis that can be confirmed with an MRI of the brain as hyperintensities in the mammillary bodies, periaqueductal area, thalami, and hippocampus. If suspected in a patient, WE must be immediately treated with intravenous thiamine to prevent evolution into Korsakoff syndrome, coma, or death. Currently, there is no consensus in the medical community as to how much thiamine must be given and for how long. Therefore, there is a need for more research in the diagnosis and management of WE after bariatric surgery. Herein, we report a rare case of a 23-year-old female with a history of morbid obesity who developed WE two weeks after a laparoscopic sleeve gastrectomy.

## Introduction

Wernicke encephalopathy (WE) is a neurological complication of vitamin B1 (thiamine) deficiency [1-4]. In 1881, neuropathologist Dr. Carl Wernicke was the first to describe this syndrome after noticing ventricular punctate hemorrhages on autopsies of chronic alcoholics [1,2,4-7]. He associated these findings with a triad of gait ataxia, confusion, and ophthalmoplegia [1-3,6,8,9]. A full triad has been reported in less than 30% of the cases, and its absence does not rule out WE [1,2,8-10]. Secondary symptoms include vestibular dysfunction, hypothermia, and peripheral neuropathy [1,4].

While WE is commonly associated with chronic alcohol use, there is an increasing number of cases in patients with no history of alcohol use [9]. This epidemiologic shift can be attributed to the growing volume of bariatric surgeries in the USA [11,12]. Bariatric surgery is an effective solution for morbid obesity [3,13,14] and is more efficacious than conservative approaches [5,15]. Micronutrient deficiencies are a common complication after bariatric surgeries, and some cases of thiamine deficiency precipitate into WE [8,15].

“Bariatric Beriberi” is a neurological emergency with a 20% mortality rate [6,8]. Thus, immediate initiation of thiamine therapy is paramount to avert residual cognitive deficits or death [13,14]. We herein present a rare case of WE in a 23-year-old female two weeks after laparoscopic sleeve gastrectomy.

## Case presentation

A 23-year-old African American female with a history of obstructive sleep apnea and morbid obesity status post-laparoscopic sleeve gastrectomy was brought to the emergency department (ED) for evaluation of hyperemesis and confusion. History obtained from the family revealed that the patient had symptoms of intermittent confusion and dysarthria/garbled speech since her sleeve gastrectomy two months prior to presentation.

Since the surgery, the patient had five to six episodes of emesis daily despite being on anti-emetic medications. She was discharged on a bariatric 1 diet (liquid diet) but did not tolerate it. The patient was also given multivitamins and vitamin B12 supplements on discharge.

Two weeks after the bariatric surgery, the patient experienced generalized weakness, blurry vision, and bilateral leg numbness up to the groins. She also had one episode of urinary incontinence a day prior to presentation but denied fecal incontinence, seizure-like activity, tongue biting, or loss of consciousness. The patient denied doing commercial weight loss diets before or after the gastric sleeve or any prior weight loss surgeries. She also denied fever, chills, headache, neck stiffness, chest pain, shortness of breath, urinary symptoms, head trauma, recent history of sick contacts, or travel.

In the ED, the patient was afebrile and saturated 98% on room air. Her vital signs were notable for a blood pressure of 144/101, heart rate of 132 beats per minute, and respiratory rate of 18 breaths per minute. The patient’s body mass index (BMI) was 52.5 kg/m^2^ before the gastric sleeve and 41.0 kg/m^2^ at this encounter. On examination, the patient was lethargic and had horizontal nystagmus. Her power was 4/5 in the upper extremities. The patient was not able to lift her legs against gravity. Her speech was coherent and intelligible when awake. The meningeal, Lhermitte, and cerebellar signs were all negative. The abdominal examination was notable for small healed surgical scars in the lower quadrants and the epigastrium. All the other systems were unremarkable.

Electrocardiogram (EKG) revealed a sinus tachycardia with T wave inversions in inferior leads and V3-V4. Troponin was normal at 10 pg/mL (normal range: 3-17 pg/mL). The admission labs revealed a white blood cell count of 20.9 x 10^3^/mm^3^ with a left shift, elevated lactic acid (3.5 mmol/L), hyperglycemia (193 mg/dL), and hypercalcemia (10.4 mg/dL). The urine toxicology screen was negative, and urinalysis showed trace leukocyte esterase and microhematuria. The patient admitted to being on her menstrual period at the time which could explain the microhematuria on the urinalysis. The patient denied dysuria or suprapubic discomfort.

A computed tomography (CT) scan of the head without contrast showed no acute bleed, mass effect, or midline shift. Due to concern for intra-abdominal infection, a CT of the abdomen and pelvis was ordered, and it was negative and only notable for hepatic steatosis. Similarly, the upper gastrointestinal series (X-ray of the upper gastrointestinal tract + abdominal radiography) was negative for obstruction or leakage. An electroencephalogram (EEG) was performed to rule out seizures, and it was negative.

**Figure 1:**
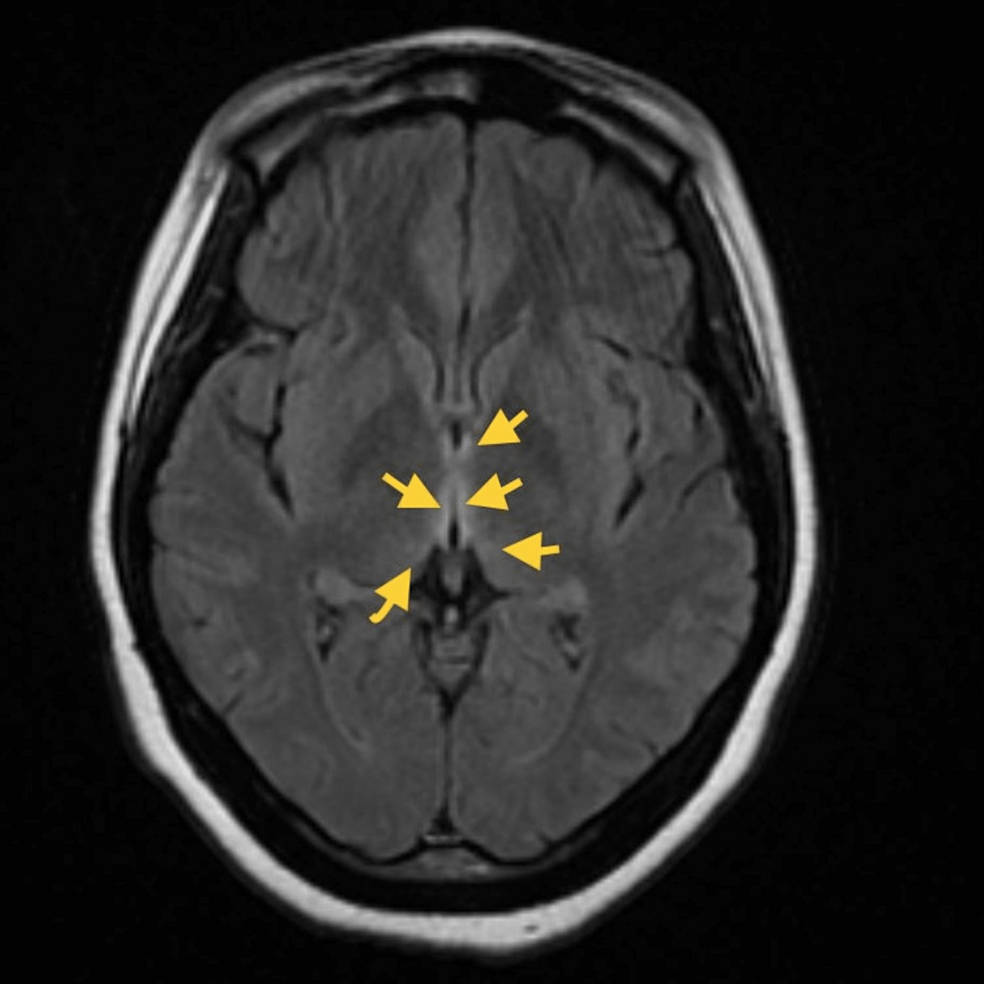
MRI of the brain without contrast FLAIR sequence MRI showing a T2 shine-through with mild edema along the hippocampus, mammillary bodies, periaqueductal gray matter, medial thalamus, and hypothalamus consistent with WE. MRI, magnetic resonance imaging; FLAIR, fluid-attenuated inversion recovery

The magnetic resonance imaging (MRI) of the brain was performed to check for infectious process, neurological pathology, or WE. No infarct was noted on imaging. The MRI (Figure 1) showed edema along the hippocampus, mammillary bodies, periaqueductal gray matter, medial thalamus, and hypothalamus, as well as the posterior hippocampus bilaterally consistent with WE.

The workup for multiple sclerosis and Wilson's disease came back negative. In the ED, the patient was empirically started on intravenous thiamine 500 mg, every 8 hours, for possible thiamine deficiency. The thiamine therapy was extended beyond three days after the diagnosis of WE was confirmed on imaging.

The patient showed improvement in mentation by day 2 and started regaining muscle strength by day 3 of thiamine supplementation. She benefitted from inpatient physiotherapy and was discharged home with a rolling walker. On discharge, she was prescribed bariatric multivitamins with minerals, calcium citrate, cholecalciferol, vitamin B12, and oral thiamine (250 mg daily). At a two-month follow-up appointment, the patient had improved significantly and had no functional deficits. She only reported occasional headaches, which improved with over-the-counter acetaminophen.

## Discussion

Bariatric surgery is an effective intervention to the ongoing obesity pandemic. This intervention is more effective and sustainable compared to the diet and exercise regimen [5,15]. Bariatric surgery is an umbrella name for surgical weight-loss procedures such as gastric banding, Roux-en-Y gastric bypass, and sleeve gastrectomy [3]. In the USA alone, the number of bariatric surgeries has increased from 160,000 in 2011 to 230,000 in 2017 [13]. Despite its many benefits, weight-loss surgery has surgical and metabolic complications [12] including micronutrient deficiencies.

Thiamine, a water-soluble vitamin, is vital for the metabolism of carbohydrates and branched-chain amino acids, and neurotransmitter synthesis [1,2,7,15,16]. Thiamine diphosphate, active form of thiamine, [9] is a co-enzyme in several steps in the Krebs cycle and pentose phosphate pathway [1]. With the body only storing 18 days of thiamine, minor deficiencies may manifest as headaches, irritability, and lethargy [6,7]. In bariatric surgery patients, only 0.0002% to 0.4% of vitamin B1 deficiency cases evolve into WE [15]. WE usually develops within the first six months post-surgery, though the presentation can be earlier if the patient was lower on thiamine before the surgery or if they have hyperemesis post-surgery [1,3].

WE, formerly known as polioencephalitis hemorrhagica superioris, is a neurological syndrome caused by thiamine deficiency [1-4,6]. The classic triad of WE includes altered mentation, gait ataxia, and ophthalmoplegia [1,2,7,8,10-14,17,18]. While the triad has withstood the test of time as diagnostic criteria, it is only present in 16-14% of the patients [2,10,17]. By chance, our patient exhibited the full triad of confusion, horizontal nystagmus, and ataxia.

If there is a high index of suspicion for WE, clinicians must begin a thiamine regimen, and diagnosis can be confirmed by resolution of symptoms [3,17]. Clinicians may also diagnose WE through a battery of ancillary tests. These include serum thiamine level, red blood cell (RBC) transketolase activity, or a T2/FLAIR magnetic resonance imaging (MRI) [17]. Urine and serum thiamine levels are unreliable and can be normal even in severe cases of WE [15].

A T2-weighted MRI can be used to confirm the diagnosis of WE or rule out other etiologies such as multiple sclerosis. A positive test is characterized by hyperintensities in the periventricular areas of the mammillary bodies, thalami, and periaqueductal regions [2,3,6-8,10-12,14]. MRI studies have a sensitivity of 50-53% and a specificity of 93% in WE diagnosis; therefore, the absence of hyperintensities is not conclusive [1,6-8,10,17]. While CTs can be considered for other differentials, they have been unremarkable in WE [2,6].

Due to the ambiguous and insidious onset of neurological symptoms, WE is often underdiagnosed [7,8]. Only around 15% of all WE cases are diagnosed antemortem [1] and even fewer cases in patients with no history of alcohol abuse [10]. WE is a neurological emergency with 20% mortality rate [6,8]. A prompt initiation of thiamine therapy may avert residual cognitive deficits, progression to Korsakoff, coma, or death [13,14]. If no clinical improvement in two to three days, other causes of acute encephalopathy must be explored and thiamine therapy be discontinued [17]. Korsakoff syndrome is the progressed form of WE and manifests as anterograde and retrograde amnesia, confabulation, and apathy [2]. Our patient was promptly started on thiamine 500 mg, IV, every 8 hours to avoid these complications. A diagnosis was confirmed three days later via an MRI, and thiamine supplementation was extended.

While clinicians agree on the empiric treatment for WE, there is no consensus as to how much thiamine should be initiated and for how long. The American Society for Metabolic and Bariatric Surgery recommends IV thiamine 200 mg, three times a day to 500 mg, daily or twice a day for three to five days, followed by 250 mg daily for three to five days or until resolution of symptoms [10]. This is then followed by 100 mg of oral thiamine daily until the patient is no longer at risk of vitamin B1 deficiency [10]. The European Federation of Neurological Societies and the Royal College of Physicians, on the other hand, recommend thiamine 500 mg, IV, three times a day until resolution of symptoms [10]. Our patient was treated with thiamine 500 mg, IV, every 8 hours for three days, followed by thiamine 250 mg, IV, for two days and discharged on 100 mg of oral thiamine daily [9,13,17].

WE in bariatric surgery patients can be avoided through preoperative nutritional assessment, proper discharge, and close follow-up after the surgery [8]. With an increasing volume of bariatric procedures in the country, clinicians must always assess for micronutrient deficiencies in patients who have undergone bariatric surgery.

## Conclusions

WE is a neurological emergency caused by vitamin B1 deficiency. While classically associated with chronic alcohol use, it has been reported to develop in patients after bariatric surgery as well. Patients often present with ataxia, confusion, and ophthalmoplegia. WE is a clinical diagnosis that is often missed because of its vague symptoms. If suspected in a patient, WE must be promptly treated to avert further complications, including death. Clinicians must consider WE as part of the differential for acute encephalopathy in patients who have undergone bariatric surgery.
